# Global challenges for seagrass conservation

**DOI:** 10.1007/s13280-018-1115-y

**Published:** 2018-11-19

**Authors:** Richard K. F. Unsworth, Len J. McKenzie, Catherine J. Collier, Leanne C. Cullen-Unsworth, Carlos M. Duarte, Johan S. Eklöf, Jessie C. Jarvis, Benjamin L. Jones, Lina M. Nordlund

**Affiliations:** 1 Seagrass Ecosystem Research Group, College of Science, Swansea University, Wallace Building, Swansea, SA2 8PP UK; 2 Project Seagrass, 33 Park Place, Cardiff, CF10 3BA UK; 3 Centre for Tropical Water & Aquatic Ecosystem Research, James Cook University, Cairns, Australia; 4 Sustainable Places Research Institute, Cardiff University, 33 Park Place, Cardiff, CF10 3BA UK; 5 Red Sea Research Center (RSRC), King Abdullah University of Science and Technology (KAUST), Thuwal, 23955-6900 Saudi Arabia; 6 Department of Ecology, Environment and Plant Sciences, Stockholm University, 106 91 Stockholm, Sweden; 7 Department of Biology & Marine Biology, Center for Marine Science, University of North Carolina Wilmington, 601 South College Rd, Wilmington, NC 28403 USA; 8 Natural Resources and Sustainable Development, NRHU Department of Earth Sciences, Uppsala University, Campus Gotland, Sweden

**Keywords:** Biodiversity, Coastal, Eelgrass, Management, Marine, Resilience

## Abstract

Seagrasses, flowering marine plants that form underwater meadows, play a significant global role in supporting food security, mitigating climate change and supporting biodiversity. Although progress is being made to conserve seagrass meadows in select areas, most meadows remain under significant pressure resulting in a decline in meadow condition and loss of function. Effective management strategies need to be implemented to reverse seagrass loss and enhance their fundamental role in coastal ocean habitats. Here we propose that seagrass meadows globally face a series of significant common challenges that must be addressed from a multifaceted and interdisciplinary perspective in order to achieve global conservation of seagrass meadows. The six main global challenges to seagrass conservation are (1) a lack of awareness of what seagrasses are and a limited societal recognition of the importance of seagrasses in coastal systems; (2) the status of many seagrass meadows are unknown, and up-to-date information on status and condition is essential; (3) understanding threatening activities at local scales is required to target management actions accordingly; (4) expanding our understanding of interactions between the socio-economic and ecological elements of seagrass systems is essential to balance the needs of people and the planet; (5) seagrass research should be expanded to generate scientific inquiries that support conservation actions; (6) increased understanding of the linkages between seagrass and climate change is required to adapt conservation accordingly. We also explicitly outline a series of proposed policy actions that will enable the scientific and conservation community to rise to these challenges. We urge the seagrass conservation community to engage stakeholders from local resource users to international policy-makers to address the challenges outlined here, in order to secure the future of the world’s seagrass ecosystems and maintain the vital services which they supply.

**Electronic supplementary material:**

The online version of this article (10.1007/s13280-018-1115-y) contains supplementary material, which is available to authorised users.

## The need for seagrass conservation

Seagrass ecosystems are of global significance to our climate and food security but remain rather unknown and on the periphery of marine conservation (Duarte et al. 2008). Ensuring healthy seagrass ecosystems around the globe will be a significant action to mitigate two of humankind’s greatest challenges: feeding and supporting the needs of more than 7 billion people and achieving some level of climate stability. Seagrasses are common and form meadows in coastal environments, typically in very shallow waters down to 60 m depth. These meadows of seagrass, monocotyledonous marine angiosperms, are globally extensive and highly productive with their distribution extending to all continents except the Antarctic. Due to their capacity to bioengineer their environment, they create a complex three-dimensional habitat in otherwise structurally limited systems which support an extensive array of biodiverse fauna. Recent estimates suggest seagrass meadows support the productivity of 20% of the world’s biggest fisheries through nursery habitat provision (Unsworth et al. 2018b). Their location in sheltered shallow waters, the rich diversity of fish and invertebrate life and the shelter they provide from predation results in an abundance of animal life. This animal life is so rich and productive that wherever they are present in proximity to human populations they form a targeted fishing ground of enormous importance to human livelihoods and well-being (Nordlund et al. 2018a; Unsworth et al. 2018b). There is also growing evidence that seagrass meadows contribute to stabilising our climate through the vast storage and sequestration of carbon within their sediments (Crooks et al. 2011; Duarte et al. 2013). Overall seagrasses play a significant role in supporting a whole range of highly valuable ecosystem services that rival those of many more illustrious and well-known ecosystems such as mangrove forests and coral reefs (Nordlund et al. 2016). For example, they form vast filters of the coastal environment (both landward and seaward), cycling nutrients and reducing bacterial pathogens capable of causing disease in humans and marine organisms (Flindt et al. 1999; Lamb et al. 2017).

Our oceans and their habitats, along with the resources and services they supply, are increasingly being subjected to anthropogenic impacts and their biodiversity and productivity are rapidly being compromised (Halpern et al. 2008; Nash et al. 2017). Ocean conservation typically focusses on the charismatic habitats and species, while ignoring many of our poorly known habitats, such as seagrass, and species that are of major significance to responding to the challenges of climate and food security. The conservation of lesser known habitats remains problematic in light of limited and finite conservation resources.

The great challenge for seagrass ecosystems is that they’re threatened globally, with evidence indicating accelerating rates of loss and degradation (Waycott et al. 2009; Unsworth et al. 2018a). Their common shallow water presence at the coastal-land interface makes them highly vulnerable to disturbance and anthropogenic impact. Given the marginalisation of these ecosystems on the world conservation agenda, understanding how we can reverse this trajectory of loss is vital. Declining coastal water quality from catchment degradation, pollutants and poor coastal zone management is placing untold pressures on seagrass ecosystems. In addition, overfishing, land reclamation, boating and aquaculture are also significant threats to seagrasses around the world (Grech et al. 2012). We need to implement effective management strategies to reverse seagrass loss and enhance their fundamental role in food provision and climate stability. In order to do this we need to understand the challenges these systems face from a multifaceted and interdisciplinary perspective, as well as identifying actions to mitigate these challenges.

Here we propose that seagrass meadows globally face a series of significant common challenges that must be addressed in order to achieve global conservation goals. We also explicitly outline a series of proposed actions that will enable the scientific and conservation community to rise to these challenges. While some of the problems outlined in our challenges are generic to many marginalised ecosystems, we believe the global nature of seagrasses, their low species diversity but high ecosystem service value, and their unique role in supporting human livelihoods mean the nature of many of these challenges are also unique to seagrass. We believe that the most significant of these challenges is a lack of societal recognition for their importance, the consequences of which are linked in at least some way to all the other challenges.

## Challenge 1: Achieving societal recognition of seagrass importance

The greatest challenge for global seagrass conservation is to enhance societal awareness of the importance of seagrass ecosystems so that bold management and restoration decisions can be met with public support (Duarte et al. 2008). Recognition of what seagrasses are and their functional contribution to human well-being remains limited in many parts of the world (Cullen-Unsworth et al. 2014). Given that seagrass is a global resource, many people have never heard of it, or they confuse it with seaweed (algae) (Jefferson et al. 2014). Where fishers depend on seagrass for livelihoods, recognition of their importance is high (Newmaster et al. 2011). But where people are removed from direct experience, or when the ecosystem service provided is indirect (e.g. the value of seagrass as a nursery ground for supporting major fisheries), recognition of what seagrass is and its importance is poor. Raising awareness is a critical challenge that addresses the widespread extent of ill-informed decisions made (from individual to government action) that contribute to continued seagrass loss and degradation.

Limited societal recognition of seagrass is exacerbated due to its apparent charisma problem in comparison with other highly charismatic habitats such as coral reefs (Duarte et al. 2008). The general public, politicians, decision makers, business leaders and all other stakeholders need to be better informed about how seagrass meadows contribute to our economies and our planetary well-being (Cullen-Unsworth et al. 2014). As the human population becomes more urban, biodiversity conservation becomes harder to achieve as a result of what has been termed the ‘extinction of experience’ (Miller 2005). People are becoming increasingly disconnected from the natural environment and direct experience with nature appears to be a prerequisite for environmental action (Dunn et al. 2006).

Wider understanding of the natural environment and its importance to people helps build social capital, increasing the tendency to change behaviour in response to environmental concerns (Pretty and Smith 2004) such as seagrass loss. Recognition by the general public does not just lead to altered personal action but can lead to pressure on policy-makers to act and empowerment of regulators to find solutions. Minimal public awareness denotes limited pressure from the public on management authorities and regulators who, as a result, are not sufficiently empowered to take action against individuals and companies responsible for seagrass damage (e.g. widespread boating damage).

To increase awareness of seagrass, we firstly propose to enhance education and experience opportunities for people of all ages. People need to experience seagrass for themselves as experiencing nature empowers people to act (Campbell et al. 1999). If we are to nurture future environmental leaders and encourage more seagrass conservation action, we need to ensure that more people have access to nature (Dunn et al. 2006). Awareness of seagrass may change with respect to locality, as research in different localities (e.g. UK vs. Tamil Nadu in India) indicates very different levels of seagrass awareness (Newmaster et al. 2011; Jefferson et al. 2014). Whether such differences exist over different levels of economic development remain unclear but studies on local ecological knowledge indicate decreasing knowledge with economic development (Pilgrim et al. 2008). As shallow intertidal to subtidal environments, seagrass habitats are highly accessible either on foot or with simple snorkelling equipment. As such they make great places to experience nature. Programmes that encourage human–nature interactions (e.g. seagrass citizen science) (Jones et al. 2018) need to be expanded to increase the opportunities for stakeholders to learn about and engage with seagrass and participate in their conservation.

**Fig. 1:**
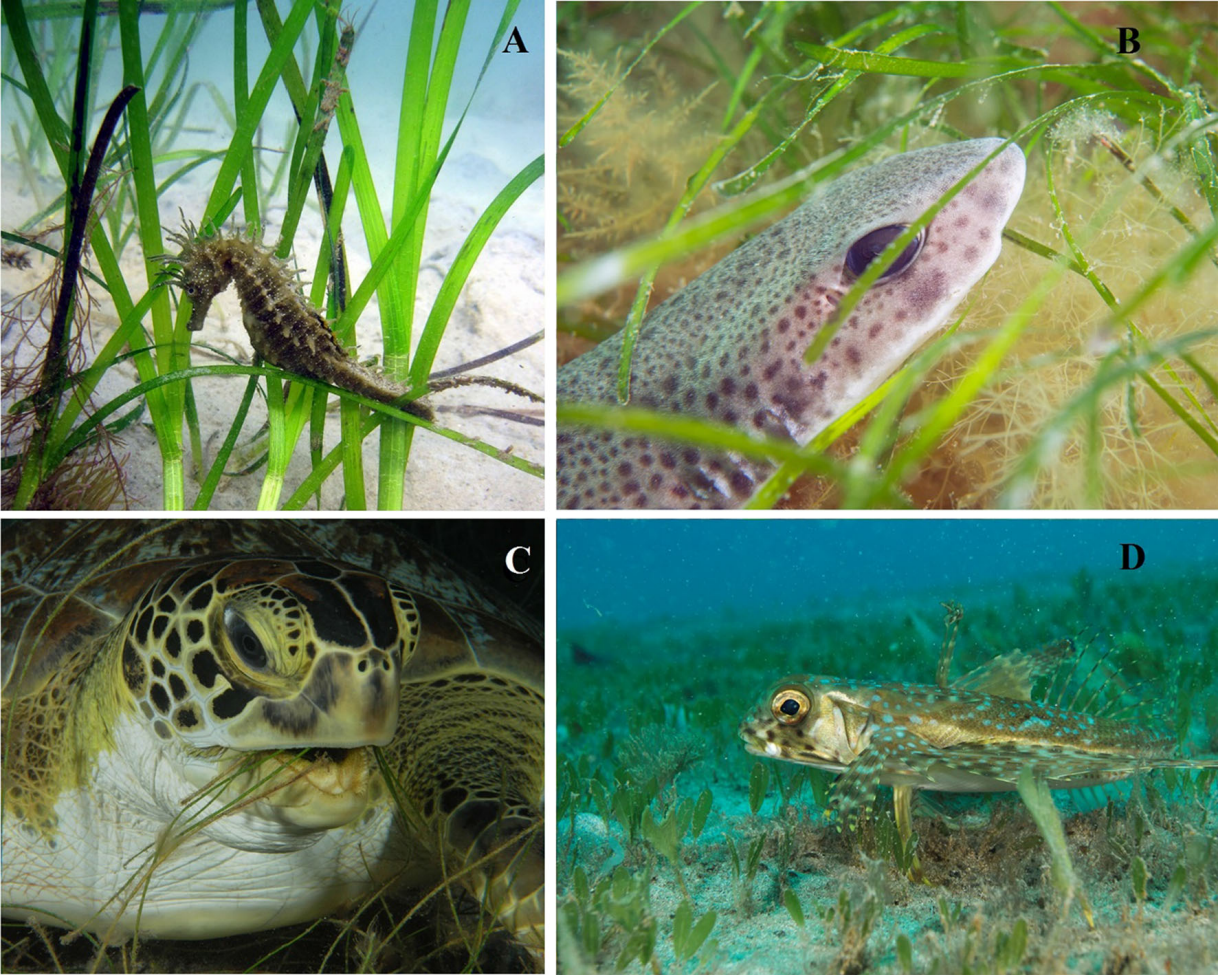
Seagrass meadows are beautiful habitats containing biodiverse faunal communities such as the following **a** the Spiny Seahorse (*Hippocampus guttulatus*) in the UK (source N Garrick-Maidment), **b** Dogfish (*Scyliorhinus canicula*) in the UK (source Frogfish Photography), **c** the Green Sea Turtle (*Chelonia mydas*) in the Dutch Antilles and **d** Flying Gurnard (*Dactylopterus volitans*) in Puerto Rico (source Luis R. Rodriguez)

Secondly, seagrass conservation needs to encompass research and experience from other fields of science communication and environmental management. For example, communicating climate science has been enhanced by adopting interdisciplinary approaches, such as inclusion of psychology and sociology to understand human reactions (Stern 2011). For example, the use of psychology could be a way of helping find a way to overcome ‘image problem’ of seagrass through the development of improved marketing and education materials. Finally, those involved with the conservation of seagrass ecosystems also need to work more closely with the global media to better highlight and communicate the value, necessity and beauty of seagrass (see Fig. 1 and Supplementary Video). Great strides have recently been made in this field through the development of the major BBC series ‘Blue Planet II’ that included extensive seagrass coverage. This progress needs to be built upon using the expanding technologies of virtual reality and 3D filming. Only through increased experiential learning opportunities, broadening conservation efforts across disciplines, and developing collaborations with global media will we be able to increase societal recognition of seagrass importance and positively impact conservation efforts.

## Challenge 2: Obtaining and maintaining information on status and condition

Global seagrass distribution and status is difficult to map and monitor, largely due to its widespread distribution and the relatively limited scientific resources focused on seagrass. Efforts to map the global distribution of seagrass appear largely stagnant, with some regions that are predicted to support vast seagrass meadows still largely uncharted (see Box 1). These data gaps provide significant challenges in regions that include a disproportionally large number of resource-poor developing countries. Documenting large-scale or deep-water seagrass distribution is challenging because of difficulties associated with reliably of detecting seagrass habitat from remote air-borne or satellite sensors, especially in complex multi-habitat seascapes, murky or deeper waters (Knudby and Nordlund 2011). For example, deep-water seagrasses in the Indian Ocean are likely extensive, yet very poorly mapped due to inaccessibility (Esteban et al. 2018). In some instances remote sensing technology can be effective to accurately resolve seagrass distribution (Kovacs et al. 2018; Phinn et al. 2018), but in many locations the only options are time-consuming and expensive field-based approaches, such as diver-, camera- or side-scan sonar-based instruments (McKenzie et al. 2001).

**Table:** 

**Box 1** Global distribution of seagrass meadows: data gaps	
Estimates of global seagrass area differ greatly throughout the literature due to limited mapping efforts and because seagrass meadows are not static, as many naturally change in distribution even in the absence of human activities. To help identify the gaps and illustrate the challenges of compiling a global seagrass resource/asset map, we used the seagrass bioregions (Short et al. 2007) to separate the global assessment into six units, based on seagrass species assemblages and geography. The documented global seagrass extent was estimated to be 325 178 km^2^ (Table 1), using the most up-to-date seagrass distribution data available as of November 2016 (see Supplementary Information). Although the Tropical Indo-Pacific has the greatest documented area (52%), the region has extensive seagrass areas not yet surveyed. Twenty countries in the Tropical Indo-Pacific region lack polygon data, which are needed to quantify seagrass meadow area, i.e. there are only point observations of seagrass occurrence. Another 42 countries are completely deficient in any data on seagrass presence (Table 1). These data gaps appear as a consequence of the large coastline and high species diversity in the Tropical Indo-Pacific region, which contains over 45 000 islands, with 17 508 in Indonesia alone. There is a pressing need to update seagrass extent data by launching new mapping campaigns. It is also likely that significantly more polygon data currently exist and a concerted effort to acquire the disparate data into a single resource is recommended. With accurate seagrass meadow maps, we can assess changes in their status enabling appropriate conservation strategies to be implemented.	

**Table 1:** Area of documented seagrass (km^2^), including number of countries, length of coastline and area of continental shelf (coastal waters to a depth of 200 m) within each of the seagrass bioregions. Number of countries with no polygon or point data is also shown. Seagrass area from polygon data as of November 2016 (for data sources see Supplementary Information), number of seagrass species from Short et al. (2007) and Short et al. (2011)

Region	Countries	Continental shelf (km^2^)	Coastline (km)	Number of seagrass species	Documented seagrass area (km^2^)	Countries lacking polygon data	Countries seagrass records absent
1. Temperate North Atlantic	25	20 015 178	207 997	5	3 430	11	7
2. Tropical Atlantic	64	2 949 362	77 804	10	109 146	17	14
3. Mediterranean	30	1 900 896	48 382	9	25 260	14	6
4. Temperate North Pacific	6	10 557 527	112 130	15	1 675	1	
5. Tropical Indo-Pacific	74	8 741 755	239 163	24	168 488	20	12
6. Temperate Southern Oceans	9	4 291 071	51 134	18	17 179	4	3
GLOBAL	208	48 455 788		72	325 178	67	42

Improved spatial assessments need to be accompanied by more widespread seagrass health and risk assessments so that early warnings of seagrass decline are available and that subsequent management measures can be taken to reverse degradation. Currently, indicators used to measure seagrass condition (e.g. biodiversity, distribution and abundance) are spatially or temporally limited. As a consequence, no globally complete database of seagrass extent or condition exists from which reference baselines can be established. Temporal data on seagrass extent are limited to sub-regional scales, for example in Denmark where nation-wide records extend for over a century (Riemann et al. 2016) and NE Australia and the western Mediterranean where only scattered decadal-scale observations are available (Rasheed and Unsworth 2011). The adoption of seagrass as a robust indicator of the health of coastal ecosystems in programmes such as the EU Water Framework Directive (Roca et al. 2016), the Chesapeake Bay (US) Program (IAN 2017) or Australia’s Great Barrier Reef Water Quality Protection Plan (McKenzie et al. 2012) have created platforms for sustained monitoring across sub-regions, but such programmes are scarce and in some cases require improvement. A significant gap in such monitoring is within developing countries that often do not have the financial resources necessary for such monitoring programmes. Such monitoring approaches need to be expanded to include all regions of the world. To rise to the challenge of mapping the world’s seagrass, first we need to rationalise disparate available data into a single resource, essentially updating the UNEP-WCMC Global Distribution of Seagrasses database (UNEP-WCMC and Short 2016), identify present data gaps and develop and standardise efficient mapping tools.

Secondly, given the extensive gaps in our global seagrass distribution database there is not a one-size-fits-all solution to this challenge. To fill these data gaps approaches need to be both top down (habitat suitability modelling and remote sensing) and bottom up (infield targeted assessments). A concerted effort, using shared reporting platforms yet to be developed, is needed from the highest levels of conservation (e.g. large international NGOs, IUCN/UNEP and governments) to the smallest of community groups. Mapping needs to harness the best features of new technology (e.g. phone apps, drones, remote sensing) as well as traditional and novel approaches (e.g. tagging of migratory seagrass-associated fauna). Furthermore, in the endeavour to map the world’s seagrasses connecting with new groups of people potentially increases the scale of available observation resources (e.g. citizen scientists) (Jones et al. 2018) and stakeholders previously not engaged in research activity (e.g. financiers involved with Blue Carbon).

Third, seagrass habitat suitability (Gattuso et al. 2006) and niche-based (Chefaoui et al. 2016) models offer major opportunities, as future mapping activity can target areas identified as potential seagrass habitats. For these models to be more effective, they require improved biophysical datasets (e.g. bathymetry and bottom substrate, seawater temperature at locations where seagrass and present and absent) that adequately represent the environmental requirements of seagrass. This requires close working of seagrass scientists with other disciplines such as oceanography to improve acquisition and availability of relevant data.

## Challenge 3: Identifying threatening activities at local scales to better target management action

There is extensive evidence of globally widespread threats to seagrass ecosystems originating from both land and ocean (Grech et al. 2012). Seagrass degradation is principally related to three broad factors: poor water quality, physical disturbance, and the degradation of food webs. Seagrass meadows provide paradigmatic examples of socio-ecological systems supporting a multitude of important ecosystem services where conservation goals and human activities often collide. Local management action targeting both direct and indirect threats is necessary (Box 2). Strategies that provide blanket protection as a key or priority habitat (Jackson et al. 2016) or assume seagrass to be a “free rider” within broader management plans incorporating other specific habitats or species are inadequate. For example, empirical evidence from the Philippines (Quiros et al. 2017) and Kenya (Eklof et al. 2009) illustrates that creating marine protected areas alone is insufficient to protect seagrass. This is because the major threats arise from land-use change. Many threats to our coastal waters, particularly those affecting water quality, originate from land, but conservation seldom includes integrated land–sea conservation planning (Nordlund et al. 2014). While some threats to seagrass are small in impact, such as the damage caused by boat moorings (Unsworth et al. 2017)s, they happen over such large scales and with such high frequency that they make seagrass highly vulnerable (Grech et al. 2012). The nature of such threats may change geographically, particularly with respect to different socio-economic circumstances (Grech et al. 2012). For example in Indonesia seagrasses are threatened by large-scale seaweed farming; such problems are typical of many developing nations within the tropics. But such threats are commonly overlooked, particularly in the presence of larger (but less frequent) or more widely acknowledged threats (e.g. water quality). It is important to consider threats that local stakeholders observe or perceive as being most persistent, and leading to higher seagrass vulnerability, to form the basis of management or conservation goals.

**Table:** 

**Box 2** The value of local understanding to seagrass threats: Case example from the Wakatobi National Park (WNP), Indonesia	
Seagrass meadows in the Wakatobi National Park (WNP), Indonesia are intensively exploited for their rich faunal communities. With a growing population and increasing fishing pressure, the area of seagrass habitat is decreasing and plant species composition and health is declining. Local ecological and expert witness knowledge was used to understand changes in seagrass area and health and to identify threats to vulnerable seagrass meadows. Multiple small but widespread and persistent impacts are described, but seagrass is considered by local stakeholders to be most vulnerable to sedimentation. Many of the impacts were considered to be exacerbated as a result of poor local appreciation for the value of seagrass. A community NGO in collaboration with scientists developed an action plan based on these findings. This resulted in two targeted conservation initiatives: (1) a widespread seagrass education programme and inclusion of seagrass in the local school curriculum; (2) an incentives programme designed to provide fruit trees to farmers and land owners to facilitate stabilisation of river banks and reduce sediment deposition to the coast. In 2017, over 4000 fruit trees were planted along three river systems, with additional communities signed up to begin the scheme. School participation was very high, paving the way for a third conservation initiative: co-development of two community-based No-fishing areas targeting seagrass meadows. This initiative exemplifies how Local Ecological Knowledge can be used to identify threats to seagrass meadows and to implement strategies to enhance conservation outcomes.	

In order to rise to the challenge of understanding local-level threats to seagrass, we believe that incorporating local ecological knowledge (LEK), including indigenous knowledge and other expert witness knowledge as alternative data sources, is key (Grech et al. 2012). Recognising the value of LEK increases stakeholder participation and commitment with management and conservation schemes (Nordlund et al. 2018b). The use of LEK can help lead to the development of spatially explicit marine conservation decision making (Grech et al. 2011) and to the creation of effective conservation management actions that form the basis of behavioural change that targets previously overlooked threats (see Box 2).

Yet, while recognising the key value of addressing local factors, evidence that seagrass ecosystems are vulnerable to ocean warming is mounting (Marba and Duarte 2010), requiring seagrass conservation efforts to extend to support efforts to mitigate climate change under the goals of the Paris Agreement, where seagrass conservation as part of blue carbon strategies is included within multiple National Declared Objectives (see Challenge 6).

## Challenge 4: Balancing the needs of people and planet

A major challenge in securing a future for seagrass meadows lies in achieving a balance between the objectives of environmental, ecological and socio-economic sustainability associated with this habitat. Seagrass meadows are recognised social-ecological systems that contribute significantly to the well-being of people and planet (Cullen-Unsworth et al. 2014). The location of seagrass meadows largely at the land to sea interface makes them extremely valuable to coastal peoples, particularly in developing regions where intertidal gleaning fisheries in seagrass can be critical for the subsistence of many people. But this positioning adds to their vulnerability where they are subject to multiple uses and multiple stressors from both land- and sea-based sources (Nordlund et al. 2014). Conflicts appear to exist between the needs of biodiversity conservation and the continued supply of seafood (Salomon et al. 2011).

We need to move away from notions considering humans as external agents of disturbance to a previously well-functioning ecosystem. We need to instead build an inclusive framework including humans as *part* of the ecosystem conducive to a sustainable and resilient future for people and planet together. The 17 Sustainable Development Goals (SDGs) of the 2030 Agenda for Sustainable Development (that superseded the Millennium Development Goals) reflect this notion and officially came into force in January 2016 (UN 2017). The SDGs apply to all countries who are expected to mobilise efforts to end poverty, fight inequality and tackle climate change. To have any chance of success, recognition in planning, monitoring and management, that ecosystems are inherently coupled social-ecological systems (SES), is the only reasonable way forward. Considering seagrass habitats within an SES framework is the most promising path to actualise successful seagrass monitoring, management and inclusion in planning processes. To do so, an integrated SES approach is needed at a variety of social and ecological scales (Hessing-Lewis et al. 2017). Sustainability is not a pseudonym for environmental conservation, as creating sustainable places requires biophysical, economic and socio-cultural sustainability at the ecosystem and increasingly at the global scale.

We need to embed conservation in a broader, multidimensional effort towards sustainable ecosystems, rather than have conservation as a stand-alone goal excluding the communities that use the ecosystems. First, we propose that to rise to the challenge of creating sustainable places we need research to expand our understanding of the interactions between the socio-economic and ecological elements of seagrass systems. This requires transdisciplinary approaches, bridging the divide between scientific disciplines (Salomon et al. 2011) and establishing encompassing partnerships with practitioners and other stakeholders (Lang et al. 2012).

Second we need data on the fishery activity in seagrass, as this is a significant component of the role of seagrass meadows as social-ecological systems (Nordlund et al. 2018a). Data are available on some commercial seagrass fisheries, but data from the collectively significant small-scale fisheries that seagrass meadows support are highly limited (Nordlund et al. 2018a). Making effective management decisions for seagrass meadows requires understanding the diversity of seagrass fisheries in terms of their economic, cultural, institutional and social values, as well as characterisation of the ecological and environmental variables. We need to rectify this in order to better target management action. We also need to improve catch monitoring of seagrass fishery activities and collect data on other fishery characteristics, in particular, fisher demographics, because too broad assumptions can lead to misguided management decisions.

Finally, we highlight that to respond to the challenge of balancing the needs of people and planet we need to recognise that seagrasses are part of a connected social-ecological system at catchment and seascape scales (de la Torre-Castro et al. 2014). Ecosystem-based management that goes beyond current geographical or habitat boundaries to encompass whole catchments needs to be more widely developed. In support of these approaches, we further need to campaign for integrated policies that seek win–win opportunities in reconciling the protection of habitats and species with the maintenance of other ecosystem services such a food security simultaneously.

## Challenge 5: Generating scientific research to support conservation actions

**Fig. 2:**
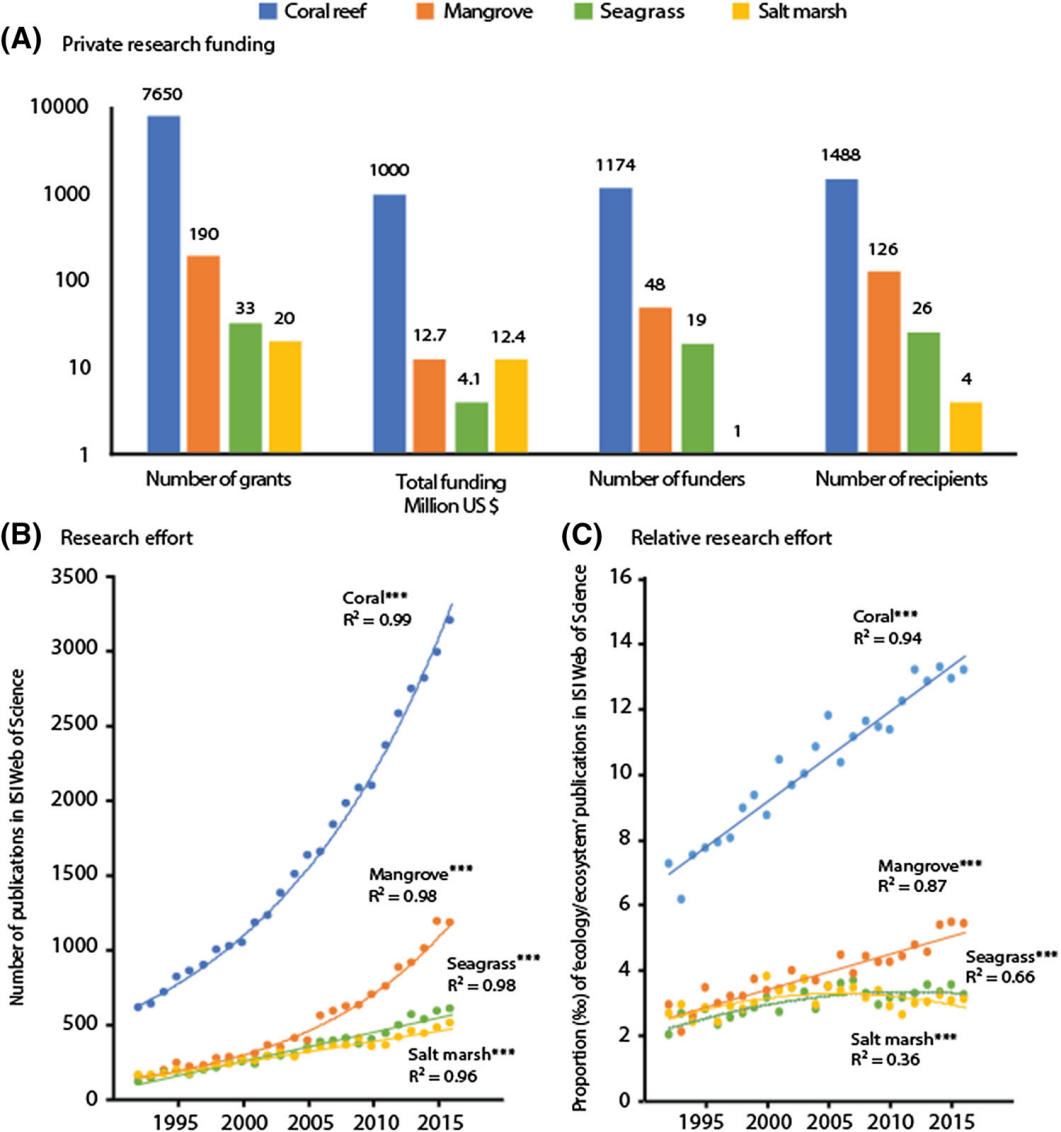
Persisting imbalance in funding to, and effort in, research and conservation on four coastal ecosystems: coral reefs, seagrass meadows, mangroves and salt marshes. Graphs show differences in **a** private foundation funding (summarised over the period 2006–2016), and increasing temporal differences in **b** research effort (number of publications per year during 1992–2016) and **c** the proportion of general ecology/ecosystem research effort (number of publications) allocated to each of the four ecosystems

A major hurdle to overcome the four challenges described above is the limited effort allocated to seagrass research and conservation, particularly when compared to other coastal and nearshore habitats. Not only is this a current problem, but we present evidence that the problem is rapidly getting worse (Box 3; Fig. 2). We suggest a major proximate cause is the fact that there are relatively few researchers studying seagrasses, particularly in relation to their widespread, near-global distribution (Hind-Ozan and Jones 2017) (see Box 3). This minimal and geographically concentrated seagrass research effort creates obvious challenges in generating and generalising research outcomes, particularly for conservation. For example, seagrass research is heavily skewed towards a few genera like *Zostera*, *Thalassia* and *Posidonia* (Nordlund et al. 2016) and for many other species we lack fundamental understanding about their distribution as well as their biology. Undoubtedly, this lack of understanding makes it difficult to manage seagrasses effectively and is exacerbated in many developing countries by funding difficulties and in some instances limited scientific capacity. Second, in many cases we do not yet understand the physical, chemical and biological attributes that combine to support the provision of different seagrass ecosystem services (Nordlund et al. 2016). Third, climate change research is perhaps one of the fastest growing sub-fields in seagrass ecology, but we still lack a predictive understanding of how global environmental change will influence seagrasses, the ecosystems they create and the services they support (see Challenge 6).

**Table:** 

**Box 3** Increasing imbalance in seagrass research funding and effort	
A decade ago, Duarte et al. (2008) (Duarte et al. 2008) demonstrated that coral reefs received far more research effort and media attention than seagrasses, mangroves and salt marshes. This is despite the wide occurrence and societal importance of all four ecosystems. Here, we show that although there has been a considerable increase in seagrass research and conservation effort, the imbalance has in fact increased over time. This involves both research funding, effort, and the proportion of general ecology and ecosystem research that this effort constitutes. First, data on private research and conservation funding 2006–2016 (retrieved from the Foundation Center database: foundationcenter.org) show that the number of grants and the total funding to grants including the word ‘coral’ exceeded those to ‘seagrass’, ‘mangrove’ and ‘marsh’ grants by 1–2 orders of magnitude (Fig. 2a). Moreover, the ‘coral’ grants were allocated to > 1 order of magnitude more recipients (researchers, practitioners, etc.). Second, data on research effort over the past 25 years (estimated as yearly number of publications in ISI Web of Science during 1992–2016) show that publications including the word ‘coral*’ in title, abstract or keywords not only dominate (Fig. 2b), but that ‘coral’ and ‘mangrove’ research effort has grown exponentially. At the same time, ‘seagrass’ and ‘salt marsh’ effort has only grown linearly and considerably slower. Finally controlling for the fact that ecology and ecosystem science in general has grown considerably (by calculating what *proportion* of yearly publications retrieved using the search string ‘ecosystem* OR ecolog* OR species*’ that also included the words ‘coral*’, ‘mangrove*’, ‘seagrass*’ or ‘salt marsh*’), a striking pattern emerges. The proportion of publications increased more or less linearly for all four ecosystems until the mid 2000s (indicating an increasing interest for and/or effort in coastal ecosystem research), after which the proportion of ‘coral’ and ‘mangrove’ research effort kept rising, but the proportion of ‘seagrass’ and ‘salt marsh’ publications instead levelled off and decreased. Together, these results suggest that seagrass (as well as salt marsh) research and conservation is underfunded, conducted by fewer people, and grows at an increasingly slower rate, than that on coral reefs (and to a lesser extent mangrove) (Fig. 2).

Seagrass conservation is also made difficult by the fact that there is often a mismatch between research funding and conservation needs. The need for additional research in support of more effective conservation might not always be obvious to decision makers or even researchers themselves. Additionally, we present data that indicate the charisma gap of seagrass ecosystems makes it difficult to find funding for seagrass research (Box 3). This is especially so for seagrass conservation-related research, particularly when competing to other, more charismatic coastal habitats. The need for novel science and the drive for researchers to publish unique findings can skew our understanding of seagrass ecosystems, e.g. by limiting local or regional study replication. Reports on simple observations of seagrass distribution, abundance and functional traits are required that are vital for making key management decisions. These data are particularly required from underrepresented areas, to avoid such biases in our knowledge. Descriptive, long-term time series of seagrasses and associated organisms, and spatially extensive surveys along natural and human-induced environmental gradients, are expensive and difficult to sustain in a research funding climate rewarding quick research output through short-term projects, but critically needed to understand seagrass ecosystem dynamics and response to local and global changes.

Wider interest in seagrass research across disciplines is growing and this needs to be built upon (Hind-Ozan and Jones 2017). First, research areas where seagrasses are already receiving considerable attention—e.g. seagrass meadows as blue carbon sinks (Fourqurean et al. 2012), the role of seagrasses for food security (Nordlund et al. 2018a; Unsworth et al. 2018b)—could be used to gain a broader interest in seagrasses from other researchers and students currently working on other systems, and motivate research about other aspects of seagrass biology. This also creates opportunities for seagrass research to integrate into wider studies about the connected coastal seascape. Second, while much seagrass research is specialised and aimed towards other specialists, there is a rapidly increasing body of high-profile studies demonstrating that the use of seagrasses as a model system to test broader questions—both fundamental and applied—can greatly increase interest in seagrasses. A few noteworthy examples include the use of seagrass to understand genome changes for angiosperms to colonise the sea (Olsen et al. 2016), the role of seagrass in removing pathogens from the water column (Lamb et al. 2017), the importance of symbiotic associations in seagrass ecosystems (van der Heide et al. 2012), the role of seagrass genetic diversity to buffer effects of disturbance (Reusch et al. 2005), and the relationship between biodiversity and ecosystem functioning (Duffy et al. 2015). While some of these studies are seagrass-specific, most target seagrasses because they constitute an ideal model system, have a relatively low (and therefore manageable) species diversity, occur along most sheltered coastlines, are relatively easy to access, and are easy to manipulate in situ, collect and grow.

Third and finally, seagrass researchers need to better communicate their research findings to a broad audience interested in marine life and the ocean, but must do so by placing seagrass as part of a wider connected seascape. Social media and online networks (e.g. Twitter, Instagram, Facebook, etc.) are simple yet powerful tools that can be used by individual researchers to spread interest in seagrasses, their ecology and their conservation to other researchers and the general public with limited effort (Hind-Ozan and Jones 2017). Although online communication tools are the necessity for science outreach in much of the globe, the internet remains a privilege of only half the worlds’ population (Sample 2018). In some nations, the use of traditional tools such as newspapers and radio may be more appropriate means of sharing scientific information than through social media.

## Challenge 6: Conservation action in an era of Climate change

Climate change is the most widespread anthropogenic threat to marine ecosystems. Direct impact to seagrass meadows include greater physical disturbance due to increasing storm frequency (Brierley and Kingsford 2009), rising water temperatures (Hyndes et al. 2016), reduced light due to sea-level rise (Grantham et al. 2011), and rising CO_2_ levels in coastal waters (Brierley and Kingsford 2009). Physiological responses to shifting environmental conditions result in species range-changes (Hyndes et al. 2016), localised invasions and extinctions (Mellin et al. 2016), and shifts in the structure and function of seagrass meadows (Björk et al. 2008). As a result, not only is the physiology of seagrass species affected by climate change but also their interactions with each other and their environment (Hyndes et al. 2016). In many regions, current legislation protecting marine resources do not expressly consider natural climate variability or anthropogenic climate change. As legislation comes up for cyclical reviews, it is important for legislative directives to move beyond focusing on mitigation (Nachmany et al. 2014) to also include adaptive and responsive mechanisms that deal directly with current and projected impacts of climate change on coastal habitats including seagrass meadows (Frost et al. 2016).

First, habitat protection policies need to incorporate projected future distributions of seagrass meadows rather than focusing on past conditions. Many species are unable to acclimate to new climate conditions, or adapt to the unprecedented pace of contemporary climate change (Collier et al. 2017). Some seagrass species, such as the Mediterranean endemic *Posidonia oceanica*, are already experiencing significant mortality with ocean warming (Marba and Duarte 2010) and are forecasted to experience dramatic losses with further warming (Jordà et al. 2012). Alternatively some seagrass species are predicted to move to track the poleward shift of isotherms (Poloczanska et al. 2013) and eventually expand into the Arctic (Krause-Jensen and Duarte 2014). In western Australia, temperate seagrasses have already contracted their ranges in response to warming ocean temperatures (Hyndes et al. 2016) and tropical ecosystems are expected to further shift their ranges as temperatures continue to warm (Vergés et al. 2014). These shifts may have significant impacts on biodiversity within seagrass meadows, especially in species rich equatorial and coastal regions (Collier et al. 2011). Researchers need to provide robust predictive models of future habitat distributions to environmental managers to enable the flexibility within policy for future changes in habitat distributions.

Secondly, seagrass monitoring should report on indicators that provide an early warning of reduced resilience, breaks in connectivity and imminent range shifts. Loss of genetic diversity affects resilience through recovery and adaptive capacity, with populations near the edge of distributional ranges being most affected (Reynolds et al. 2016). Therefore, a loss of genetic diversity could be an early-warning indicator of loss of resilience and the potential for range contraction, particularly when it is impractical to monitor distributional ranges. Quantifying the density and viability of seagrass seed banks can also provide a measure of seagrass resilience as germination of seeds and the development of seedlings provides a recovery mechanism following large-scale declines (Jarvis and Moore 2010). Finally, efforts must be made to conserve connectivity between populations and to conserve links between source and sink populations for seagrass propagules (McMahon et al. 2014) including the protection of biological dispersal agents (e.g. megaherbivores Tol et al. 2017). One possibility would be to move away from static MPAs to connectivity-informed MPAs that are spatially designed to maximise connectivity between source and sink populations or dynamic MPAs that adjust for changing species distributions.

Thirdly, environmental targets that are set for seagrass conservation (e.g. water quality guidelines) should move towards future climate adjusted targets and allow for cumulative impacts and ecological feedbacks (Maxwell et al. 2017). For example, elevated water temperature increases seagrass light requirements (Collier et al. 2012) and both elevated temperature and low light create a negative feedback at the sediment-plant scale (Koch et al. 2007). If these interactions and feedbacks are quantified, they can be accommodated in water quality guidelines. Similarly, the timing of other anthropogenic disturbances can be managed to avoid unnecessary cumulative impacts, e.g. dredging at a time of greatest risk from extreme temperature (Wu et al. 2017). Sub-lethal indicators with a distinct cause–effect pathway can also provide ‘real-time’ feedback when environmental targets are breached (McMahon et al. 2013). These early-warning signs can enable management prioritisation and set associated achievable management goals to minimise the risk of cumulative impacts including climate change.

Finally, active intervention strategies such as innovative restoration techniques will be increasingly required to repair ecological function following disturbances, and need to be adaptable to changing climatic conditions (Timpane-Padgham et al. 2017). Changes in flowering effort, frequency and timing within species lifecycles in response to warming temperatures have also already been observed for seagrasses around the world (Jarvis et al. 2011; Suonan et al. 2017). These heat-adapted populations may provide suitable restoration propagules for those that do not have heat resistance, but face increasing temperatures. However, not all species have shown potential for natural acclimation (Collier et al. 2017), in which case additional strategies may be required to maintain ecological function.

## Conclusion

To secure the future of the world’s seagrass ecosystems, we need to respond to the six global challenges outlined here with actions (see Box 4 summary). These actions may differ with respect to their means of application in different parts of the globe (e.g. developed vs developing nations) but we believe these challenges reflect the global needs of seagrass conservation. Many of these responses necessitate improved interdisciplinary and multidisciplinary science and conservation in order to facilitate a fundamental shift in the recognition and management of seagrass meadows. Although progress is being made to conserve seagrass meadows in some locations, there are meadows of major global significance that remain on a downward trajectory, and many meadows whose importance as well as status remains unknown. Conservation and communication need to be supercharged across planning scales from local communities to international policy-makers. The expectation that seagrass meadows will continue supporting food security, mitigating climate change and supporting biodiversity will only be realised if we rise to these challenges without delay.

**Table:** 

**Box 4** Summary of the six challenges for seagrass conservation and proposed policy responses	
**Challenge 1: Societal recognition of seagrass importance** (1) General public needs to experience seagrass for themselves. (2) Seagrass conservation needs to expand focus to encompass research and experience. (3) Expand work with the global media. **Challenge 2: Up-to-date information on status & condition** (1) Rationalise disparate available global data into a single resource. (2) Improved top-down (habitat suitability and niche modelling and remote sensing) and bottom-up (infield targeted assessments) data collection. **Challenge 3: Identifying threatening activities at local scales to target management actions accordingly** (1) Harness local ecological knowledge (LEK) to gather information in data poor areas. **Challenge 4: Balancing the needs of people and planet** (1) Expand understanding of interactions between the socio-economic and ecological elements of seagrass systems. (2) Data required on the fishery activity in seagrass. (3) Recognise seagrasses as part of connected social-ecological system at catchment and seascape scales. **Challenge 5: Generating scientific research to support conservation actions** (1) Use current high-profile seagrass research (food security and blue carbon) to engage wider research fields. (2) Encourage use of seagrass as a model ecological system or model species. (3) Improved and increased communication of research to a broad audience. **Challenge 6: Conservation action in an era of Climate change** (1) Incorporate projected future distribution into habitat protections. (2) Use of indicators that provide an early warning of seagrass climate change impacts. (3) Use future climate adjusted conservation targets that allow for cumulative impacts and ecological feedbacks. (4) Develop innovate restoration techniques.	

## Electronic supplementary material

Below is the link to the electronic supplementary material.

Supplementary material 1 (PDF 87 kb)

Supplementary material 2 (MP4 20490 kb)
