# Supplementary material for: Global challenges for seagrass conservation
Source: Ambio. 2018 Nov 19;48(8):801–15. doi: 10.1007/s13280-018-1115-y (PMC6541581; doi:10.1007/s13280-018-1115-y)
Supplement: Supplementary file 1 — Supplementary material 1 (PDF 87 kb) [file 13280_2018_1115_MOESM1_ESM.pdf]

Ambio

Electronic Supplementary Material

Title: **Global challenges for seagrass conservation**

Authors: Richard K.F. Unsworth, Len J McKenzie, Catherine J. Collier, Leanne C. Cullen-Unsworth, Carlos M. Duarte, Johan S. Eklöf, Jessie C. Jarvis, Benjamin L. Jones, Lina M. Nordlund

Supporting Information

S 1. Information sources used to calculated current documented global area of seagrass

Seagrass meadows range in size from a few square meters to thousands of square kilometres. Maps of seagrass distribution are either individual observations (points) or measured areas (polygons or vector-based). As polygon maps provide the only quantitative measure of spatial extent, they were used exclusively for areal estimates. Point data was used only to indicate seagrass presence.

The most extensive collection of seagrass maps is located at the UNEP World Conservation Monitoring Centre ((UNEP-WCMC and Short 2016)). This collection includes maps from various times, of various scales (ranging from 1:1,000 ((Telesca et al. 2015)) to 1:28,510,000 ((National Geographic Society 2000))),from variable sources, and ranging from anecdotal (no documented/visual evidence) to accurate field validation ((UNEP-WCMC and Short 2016)). Although detailed, this collection remains incomplete, as not all researchers and/or agencies contribute data. For our estimate of global seagrass area we have used UNEP-WCMC and Short ((2016)) and where available, included additional polygon data from published literature. Where possible, we have also corrected some of the anecdotal polygon data.

Table S1: Data sources for documented seagrass polygon area (km^2) data (as of November 2016) for each seagrass bioregion.

Seagrass Bioregion	Data Source
1. Temperate North Atlantic	(Boström et al. 2014, Cunha et al. 2014, UNEP-WCMC and Short 2016, Wilkes et al. 2017)
2. Tropical Atlantic	(Green and Short 2003, Murdoch et al. 2007, DaCosta-Cottam et al. 2009, Krupp et al. 2009, Ballhorn et al. 2014, Samper-Villarreal et al. 2014, Creed et al. 2016, UNEP-WCMC and Short 2016)
3. Mediterranean	(Deyanova et al. 2013, UNEP-WCMC and Short 2016)
4. Temperate North Pacific	(Green and Short 2003, CEC 2016, UNEP-WCMC and Short 2016)
5. Tropical Indo-Pacific	(ICMAM PD 2001, Bujang and Zakaria 2003, Green and Short 2003, Orosco and Amir Sharifudeen 2004, McKenzie and Rasheed 2006, BAE Systems 2007, UNEP 2007, Fortes 2008, Vibol 2008, Boggs et al. 2009, Coles et al. 2009, Kamal and Khan 2009, Sridhar et al. 2010, Waycott et al. 2011, Boggs et al. 2012, Luong et al. 2012, Nobi et al. 2012, Susila et al. 2012, Nobi et al. 2013, Yaakub et al. 2013, McKenzie et al. 2014, Marine and Mangrove Research and Development Center 2015, Rajamani and Marsh 2015, McKenzie et al. 2016, Poonian et al. 2016, UNEP-WCMC and Short 2016, Cuvillier et al. 2017)
6. Temperate Southern Oceans	(Poiner et al. 1987, Green and Short 2003, Roelofs et al. 2005, UNEP-WCMC and Short 2016)

S 2. Method used to examine changes in research effort and grant funding for seagrass over time.

First, data on private research and conservation funding 2006-2016 (retrieved from the Foundation Center database: foundationcenter.org) shows that the number of grants and the total funding to grants including the word ‘coral’ exceeded those to ‘seagrass’, ‘mangrove’ and ‘marsh’ grants by 1-2 orders of magnitude (Fig. 1A). Moreover, the ‘coral’ grants were allocated to more recipients

(researchers, practitioners, etc.) by >1 order of magnitude. Second, data on research effort over the past 25 years (estimated as yearly number of publications in ISI Web of Science during 1992-2016) shows that publications including the word 'coral*' in title, abstract or keywords not only dominate (Fig 1B), but that 'coral' and 'mangrove' research effort has grown exponentially. At the same time, 'seagrass' and 'salt marsh' effort has only grown linearly and considerably slower. Finally controlling for the fact that ecology and ecosystem science in general has grown considerably (by calculating what proportion of yearly publications retrieved using the search string 'ecosystem* OR ecolog* OR species*' that also included the words 'coral*', 'mangrove*', 'seagrass*' or 'salt marsh*') a striking pattern emerges. The proportion of publications increased more or less linearly for all four ecosystems until the mid 2000s (indicating an increasing interest for and/or effort in coastal ecosystem research), after which the proportion of 'coral' and 'mangrove' research effort kept rising, but the proportion of 'seagrass' and 'salt marsh' publications instead levelled off and decreased.

Sources:

BAE Systems. 2007. Mapping of Benthic Habitats for The Main Eight Hawaiian Islands: Task Order 1 Project Completion Report. BAE Systems Sensor Solutions Identification & Surveillance (S2 IS), Honolulu, HI.

Ballhorn, U., C. Mott, and F. Sieger. 2014. Establishing the baseline for seagrass and mangrove area cover in four Marine and Coastal Priority Protected Areas within the Meso-American Reef area: Punta de Manabique Wildlife Refuge, Guatemala. Remote Sensing Solutions, München, Germany.

Boggs, G., K. Edyvane, N. de Carvalho, S. Penny, J. Rouwenhorst, P. Brocklehurst, I. Cowie, C. Barreto, A. Amaral, J. Monteiro, P. Pinto, R. Mau, N. Smit, J. Amaral, and L. Fernandes. 2012. Marine and Coastal Habitat Mapping in Timor Leste (North Coast) – Final Report. Project 1 of the Timor Leste Coastal-Marine Habitat Mapping, Tourism and Fisheries Development Project. Ministry of Agriculture & Fisheries, Government of Timor Leste, Dili, Timor Leste.

Boggs, G., K. Edyvane, N. de Carvalho, S. Penny, J. Rouwenhorst, P. Brocklehurst, I. Cowie, C. Barreto, A. Amaral, N. Smit, J. Monteiro, P. Pinto, R. Mau, J. Amaral, and L. Fernandes. 2009. Marine and Coastal Habitat Mapping in Timor Leste (North Coast) – Final Report. Ministry of Agriculture & Fisheries, Government of Timor Leste, Dili, Timor Leste.

Boström, C., S. Baden, A.-C. Bockelmann, K. Dromph, S. Fredriksen, C. Gustafsson, D. Krause-Jensen, T. Möller, S. L. Nielsen, B. Olesen, J. Olsen, L. Pihl, and E. Rinde. 2014. Distribution, structure and function of Nordic eelgrass (Zostera marina) ecosystems: implications for coastal management and conservation. Aquatic Conservation **24**:410-434.

Bujang, J. S., and M. H. Zakaria. 2003. The seagrasses of Malaysia. Pages 152-160 *in* E. Green and F. Short, editors. World Atlas of Seagrasses. University of California Press, Berkeley. USA.

CEC. 2016. North America's Blue Carbon: Assessing Seagrass, Salt Marsh and Mangrove Distribuition and Carbon Sinks. Commission for Environmental Cooperation (CEC), Montreal, Canada.

Coles, R., L. J. McKenzie, G. De'ath, A. Roelofs, and W. J. Lee Long. 2009. Spatial distribution of deepwater seagrass in the inter-reef lagoon of the Great Barrier Reef World Heritage Area. Marine Ecology Progress Series **392**:57-68.

Creed, J. C., A. H. Engelen, E. C. D´Oliveira, S. Bandeira, and E. A. Serrão. 2016. First record of seagrass in Cape Verde, eastern Atlantic. Marine Biodiversity Records **9**:57.

Cunha, A. H., J. F. Assis, and E. A. Serrão. 2014. Reprint of "Seagrasses in Portugal: A most endangered marine habitat". Aquatic Botany **115**:3-13.

Cuvillier, A., N. Villeneuve, E. Cordier, J. Kolasinski, L. Maurel, N. Farnier, and P. Frouin. 2017. Causes of seasonal and decadal variability in a tropical seagrass seascape (Reunion Island, south western Indian Ocean). Estuarine, Coastal and Shelf Science **184**:90-101.

DaCosta-Cottam, M., J. Olynik, J. Blumenthal, K. D. Godbeer, J. Gibb, J. Bothwell, F. J. Burton, P. E. Bradley, A. Band, T. Austin, P. Bush, B. J. Johnson, L. Hurlston, L. Bishop, C. McCoy, G. Parsons, J. Kirkconnell, S. Halford, and G. Ebanks-Petrie. 2009. Cayman Islands National Biodiversity Action Plan 2009. Cayman Islands Government. Department of Environment, George Town, Grand Cayman.

Deyanova, D., D. Berov, and V. Karamfilov. 2013. Seagrass Habitats Distribution and Ecological State Along the Southern Bulgarian Black Sea Coast. MARES 2020 International Conference "Marine Research Horizon 2020", 17-20 September 2013, Varna, Bulgaria.

Fortes, M. 2008. National report on seagrass in the South China Sea – Philippines. Page 20 *in* UNEP, editor. National reports on seagrass in the South China Sea. UNEP/GEF/SCS Technical Publication No. 12. United Nations Environment Programme, Bangkok, Thailand.

Green, E., and F. Short, editors. 2003. World Atlas of Seagrasses. University of California Press,, Berkeley. USA.

ICMAM PD. 2001. Resources Information System for Gulf of Mannar (India). Integrated Coastal and Marine Area Management Project Directorate (ICMAM PD), Department of Ocean Development, Chennai, India.

Kamal, A. H. M., and M. A. A. Khan. 2009. Coastal and estuarine resources of Bangladesh : management and conservation issues. Maejo Int. J. Sci. Technol. **3**:313-342.

Krupp, L. S., J. Cortés, and M. Wolff. 2009. Growth dynamics and state of the seagrass Thalassia testudinum in the Gandoca-Manzanillo National Wildlife Refuge, Caribbean, Costa Rica. Revista de Biología Tropical **57**:187-201.

Luong, C. V., N. V. Thao, T. Komatsu, N. D. Ve, and D. D. Tien. 2012. Status and threats on seagrass beds using GIS in Vietnam. Page 13 *in* SPIE Asia-Pacific Remote Sensing. SPIE.

Marine and Mangrove Research and Development Center. 2015. Report on the assessment and status of mariine and coastal resources: coral and seagrass. Department of Marine and Coastal Resources, Ministry of Natural Resources and Environment, Agricultural Cooperatuve Federation of Thailand, Thailand.

McKenzie, L. J., S. J. Campbell, and F. Lasi. 2016. Seagrass meadows of the Solomon Islands, derived from field surveys conducted 13 May and 16 June 2004. PANGAEA.

McKenzie, L. J., and M. J. Rasheed. 2006. Seagrass meadows of Pohnpei and And Atoll, Federated States of Micronesia, derived from field surveys conducted 26 October-03 November, 2005. PANGAEA.

McKenzie, L. J., R. L. Yoshida, A. Grech, and R. Coles. 2014. Composite of coastal seagrass meadows in Queensland, Australia - November 1984 to June 2010. . PANGAEA:http://doi.pangaea.de/10.1594/PANGAEA.826368.

Murdoch, T. J. T., A. F. Glasspool, M. Outerbridge, J. Ward, S. Manuel, J. Gray, A. Nash, K. A. Coates, J. Pitt, J. W. Fourqurean, P. A. Barnes, M. Vierros, K. Holzer, and S. R. Smith. 2007. Large-scale decline in offshore seagrass meadows in Bermuda. Marine Ecology Progress Series **339**:123-130.

National Geographic Society. 2000. Coral World. National Geographic Society, Washingon, D.C.

Nobi, E. P., E. Dilipan, K. Sivakumar, and T. Thangaradjou. 2012. Estimation of the Aerial Cover of Seagrasses of Lakshadweep Islands (India) Using Indian Remote Sensing Satellite (IRS P6 LISS IV). Journal of the Indian Society of Remote Sensing **40**:467-481.

Nobi, E. P., E. Dilipan, T. Thangaradjou, and P. K. Dinesh Kumar. 2013. Restoration scaling of seagrass habitats in the oceanic islands of Lakshadweep, India using geospatial technology. Applied Geomatics **5**:167-175.

Orosco, C., and A. Amir Sharifudeen. 2004. The distribution of the seagrass, *Halophila beccarii* Aschers., in Terengganu River estuary. Pages 192-195 *in* KUSTEM Third Annual Seminar on Sustainability Science and Management 2004 : role of environmental science and technology in sustainable development of resources, 4-5 May, 2004. Penerbit Kolej Universiti Sains dan Teknologi Malaysia, Primula Beach Resort, Kuala Terengganu, Terengganu.

Poiner, I. R., D. J. Staples, and R. Kenyon. 1987. Seagrass communities of the Gulf of Carpentaria, Australia. Australian Journal of Marine and Freshwater Research **38**:121-131.

Poonian, C. N. S., K. Tuharska, and M. D. Hauzer. 2016. Diversity and distribution of seagrasses in the Union of the Comoros. African Journal of Marine Science **38**:263-268.

Rajamani, L., and H. Marsh. 2015. Mapping seagrass cost-effectively in the Coral Triangle: Sabah, Malaysia as a case study. Pacific Conservation Biology **21**:113-121.

Roelofs , A. J., R. G. Coles, and N. Smit. 2005. A survey of intertidal seagrass from Van Diemen Gulf to Castlereagh Bay, Northern Territory, and from Gove to Horn Island, Queensland. Report to the National Oceans Office., Department of Primary Industries & Fisheries, Cairns.

Samper-Villarreal, J., A. Bourg, J. A. Sibaja-Cordero, and J. Cortés. 2014. Presence of a Halophila baillonii Asch. (Hydrocharitaceae) Seagrass Meadow and Associated Macrofauna on the Pacific Coast of Costa Rica. Pacific Science **68**:435-444.

Sridhar, R., T. Thangaradjou, L. Kannan, and S. Astalakshmi. 2010. Assessment of coastal bio-resources of the Palk Bay, India, using IRS-LISS-III data. Journal of the Indian Society of Remote Sensing **38**:565-575.

Susila, S., E. Nobi, T. Thangaradjou, K. Sivakumar, P. Anantharaman, L. Kannan, and T. Balasubramanian. 2012. *In situ* biomass estimation of seagrasses in the Gulf of Mannar (India) and their areal coverage using

IRS P6 LISS III. Pages 209–226 *in* K. Sridhar, editor. Aquatic Plants and Plant Diseases: Types. Characteristics and Management. NOVO Science Publishers, New York.

Telesca, L., A. Belluscio, A. Criscoli, G. Ardizzone, E. T. Apostolaki, S. Fraschetti, M. Gristina, L. Knittweis, C. S. Martin, G. Pergent, A. Alagna, F. Badalamenti, G. Garofalo, V. Gerakaris, M. Louise Pace, C. Pergent-Martini, and M. Salomidi. 2015. Seagrass meadows (Posidonia oceanica) distribution and trajectories of change. Scientific Reports **5**:12505.

UNEP-WCMC, and F. Short. 2016. Global distribution of seagrasses (version 4.0). Fourth update to the data layer used in Green and Short (2003). UNEP World Conservation Monitoring Centre, Cambridge (UK).

UNEP. 2007. Procedure for Determination of National and Regional Economic Values for Ecotone Goods and Services, and Total Economic Values of Coastal Habitats in the context of the UNEP/GEF Project Entitled: "Reversing Environmental Degradation Trends in the South China Sea and Gulf of Thailand". South China Sea Knowledge Document No. 3. UNEP/GEF/SCS/Inf.3. United Nations Environment Programme, Bangkok, Thailand.

Vibol, O. 2008. National report on seagrass in the South China Sea – Cambodia. Page 14 *in* UNEP, editor. National reports on seagrass in the South China Sea. UNEP/GEF/SCS Technical Publication No. 12. United Nations Environment Programme, Bangkok, Thailand.

Waycott, M., L. J. McKenzie, J. E. Mellors, J. C. Ellison, M. T. Sheaves, C. Collier, A.-M. Schwarz, A. Webb, J. Johnson, and C. E. Payri. 2011. Vulnerability of mangroves, seagrasses and intertidal flats in the tropical Pacific to climate change. Pages 97-168 *in* J. D. Bell, J. E. Johnson, and A. J. Hobday, editors. Vulnerability of fisheries and aquaculture in the Pacific to climate change. Secretariat of the Pacific Community, Noumea, New Caledonia.

Wilkes, R., M. Bennion, N. McQuaid, C. Beer, G. McCullough-Annett, K. Colhoun, R. Inger, and L. Morrison. 2017. Intertidal seagrass in Ireland: Pressures, WFD status and an assessment of trace element contamination in intertidal habitats using Zostera noltei. Ecological Indicators **82**:117-130.

Yaakub, S. M., R. L. F. Lim, W. L. Lim, and P. A. Todd. 2013. The diversity and distribution of seagrass in Singapore. Nature in Singapore **6**:105–111.
